# A randomized trial comparing supplemental video care and asynchronous remote monitoring among young people with type 1 diabetes and elevated glycemic levels

**DOI:** 10.48130/pedi-0026-0007

**Published:** 2026-08-26

**Authors:** Stephanie Crossen, Salvador Lopez, Carrie Lewis, Daniel Tancredi, Amy Hughes-Lansing, Nicole Glaser

**Affiliations:** 1 Department of Pediatrics, University of California Davis School of Medicine, Sacramento, CA 95817, USA; 2 Center for Healthcare Policy and Research, University of California Davis, Sacramento, CA 95817, USA; 3 Department of Psychological Science, University of Vermont, Burlington, VT 05401, USA

**Keywords:** Type 1 diabetes, Telehealth, Remote patient monitoring, Digital health

## Abstract

Telehealth can enable supplemental care to young people with type 1 diabetes (T1D) in need of frequent guidance or treatment changes. However, the relative benefits of different telehealth modalities (e.g., synchronous video visits versus asynchronous remote patient monitoring [RPM]) for specific populations remain unclear. We conducted a three-arm randomized controlled trial comparing supplemental monthly video visits or monthly RPM versus usual care over 6 months among young people aged 5−18 years with established T1D and hemoglobin A1c (HbA1c) > 8%. The study cohort of 65 participants was 57% publicly insured, with 54% White and 26% Hispanic/Latino, a mean age of 13.4 years, a T1D duration of 5.8 years, a baseline HbA1c of 9.7%, and high baseline use of continuous glucose monitoring (98%) and insulin pumps (77%). Both study arms experienced high rates of attrition (45% among video participants, 35% among RPM participants). Compared with RPM encounters, video visits were longer (mean: 29.8 vs 17.2 min, *p* < 0.001) and more likely to cover topics beyond insulin dose adjustments, such as diabetes behaviors, technology use, and emotional support. Intention-to-treat analysis demonstrated a trend (*p* = 0.06) toward 1.0% lower mean HbA1c at study completion among video participants compared with usual care, adjusting for baseline factors; no significant difference was seen for RPM. Feedback about both interventions was highly positive. These findings suggest that among young people with elevated HbA1c despite using diabetes technology, supplemental synchronous, individualized interactions may be more effective at reducing HbA1c than supplemental data review with asynchronous outreach.

## Introduction

The Diabetes Control and Complications Trial (DCCT) demonstrated that intensive insulin therapy combined with frequent clinical contact was a recipe for optimal glycemic management in people with type 1 diabetes (PwT1D)^[1]^. In the decades since, insulin therapy has evolved dramatically from multiple daily injections to automated insulin delivery systems^[2]^, but our knowledge about the ideal frequency and modality of clinical outreach for PwT1D remains insufficient. Guidelines maintain the importance of quarterly office visits,^[3]^ but research shows that young people with elevated hemoglobin A1c (HbA1c) levels benefit from more frequent encounters^[4]^. Leveraging this type of supplemental care may be an important avenue for closing existing disparities in diabetes outcomes for marginalized populations^[5]^.

The advent of home-to-clinic telehealth and the ability to share glucose data remotely have made more frequent clinical encounters feasible from a patient standpoint. However, questions remain for clinicians and health systems about how best to deliver supplemental care for PwT1D using limited resources, such as the optimal timing, modality, and content of clinician outreach. Our previous trial of supplemental monthly video visits in young PwT1D and elevated HbA1c demonstrated significant improvements in HbA1c over 12 months (0.8% among all participants and 1.2% among those who completed the full year)^[4]^. Similarly, our pilot study of monthly remote patient monitoring (RPM) among young PwT1D using continuous glucose monitoring (CGM) showed a 0.68% improvement in glucose management indicators (GMIs) after 6 months among participants with baseline HbA1c ≥ 8%.^[6]^ Participant feedback from these studies suggested that more frequent contact was beneficial for maintaining focus on diabetes self-management, and that specific input about insulin dose adjustments as well as daily diabetes behaviors and how to optimize the use of diabetes technology were particularly helpful.

Given these preliminary findings, we conducted a randomized controlled trial to evaluate the relative effects of supplemental monthly video visits or monthly RPM over a 6-month period among pediatric patients with T1D and elevated glycemic measures (HbA1c or GMI ≥ 8%). Our primary aim was to assess the impact of these interventions on glycemic levels (the primary outcome), as well as use of diabetes technology, healthcare utilization, and patient-reported outcomes (PRO), including diabetes-related distress and self-efficacy (secondary outcomes). The secondary aims were to collect pertinent process-based measures (e.g., outreach content and time duration) and participants' feedback (e.g., perceived benefits and burdens, preferences for future care) for these two types of supplemental care. We hypothesized that both supplemental video visits and supplemental RPM would result in improved HbA1c after 6 months compared with usual care, that video visits would improve diabetes technology use and diabetes-related self-efficacy compared with usual care, and that participants would prefer the convenience of RPM to scheduled video visits.

## Methods

### Enrollment

Participants were recruited from the UC Davis Pediatric Diabetes Clinic between September 2022 and November 2023. This clinic serves approximately 800 young PwT1D and provides multidisciplinary diabetes care through a combination of office visits and video visits, typically scheduled at quarterly intervals with more frequent follow-up on an as-needed basis.

Inclusion criteria for this study were: (1) age 5−18 years, (2) T1D duration of ≥ 12 months, (3) at least one elevated glycemic measure (defined as HbA1c ≥ 8%, 14-day GMI ≥ 8% from CGM data, or 14-day average blood glucose ≥ 200 mg/dL) in the preceding months, (4) intention to receive diabetes care at the UCD Pediatric Diabetes Clinic during the following year, (5) access to the internet via a device with video and audio capability, and (6) the ability to connect the patient's home glucose meter or CGM device, as well as their insulin pump, if applicable, to an internet-capable device via Bluetooth or a physical cable.

Patients were excluded from participation if they lived outside of California because of physician licensing restrictions around interstate telehealth services. Written informed consent was obtained from participants 18 years of age or older. For patients under 18 years of age, parental written informed consent was obtained, and the patients' verbal assent was also obtained for those 8–17 years of age. The UC Davis Institutional Review Board reviewed and approved this study protocol, and this study is registered at clinicaltrials.gov (NCT04428658).

Before randomization, each participant completed a test run of remote data sharing and a home-to-clinic video encounter with the research staff to resolve any technical issues. Participants who successfully completed the test run were randomized to one of three study arms: Usual care, supplemental video visits, or supplementary RPM.

Randomization was completed via a computer-generated permuted block randomization scheme with variable block sizes, generated by the statistician and uploaded into REDCap such that assignments were concealed from the study team until the time of randomization. Allocations were initially 1:1:1 into the three study arms. However, one year after the initiation of recruitment, it was clear that the two intervention arms were experiencing higher attrition rates than the usual care arm, so the randomization scheme at that time was changed to 2:2:1 (video:RPM:usual care) from that point forward to optimize our ability to collect data about the interventions. This change was made without a review of any outcome data, and both the UC Davis Institutional Review Board and the clinical trial registry were updated about this change.

### Intervention

Participants in the usual care arm continued to receive multidisciplinary care at our clinic as recommended by their diabetes provider(s). Participants in the video arm received their usual care plus monthly video visits with a study physician (S.C.). Video visits were conducted with both the patient and caregiver, and included a discussion of interval health events, a review of shared glucose data and insulin dose information, and provision of recommendations by the physician. Participants in the RPM arm received their usual care plus monthly RPM by a study physician (S.C.) who reviewed their glucose data (and pump data, if available) asynchronously and contacted them via a secure text platform and/or telephone to provide individualized recommendations.

Recommendations provided for participants in both intervention arms (video and RPM) could include insulin dose changes, use of diabetes technology, and/or strategies for diabetes-related behaviors and family communication, depending on the needs of the individual. Insulin dose adjustments were made at the discretion of the study endocrinologist according to her clinical judgment, as would occur during a typical diabetes visit or asynchronous care discussion using remotely shared data. Interventions were delivered monthly up to a total of six times (or fewer if participants chose to stop receiving the intervention or were unresponsive to study team outreach).

### Data collection

At the time of enrollment, demographic information and baseline variables related to diabetes care (e.g., date of diagnosis, use of diabetes technology) were extracted from the electronic health record (EHR) in order to characterize the study population. Baseline HbA1c was recorded as the most recent HbA1c within the month leading up to enrollment (for most participants, this was done on the day of enrollment during a concurrent diabetes care encounter). If an HbA1c reading was not available in the preceding month (e.g., in circumstances of remote recruitment during a telehealth visit), a home HbA1c kit was mailed to the participant, with the results obtained prior to randomization.

Two validated psychosocial instruments, namely the Self Efficacy in Diabetes Management (SEDM)^[7]^ and Problem Areas in Diabetes (PAID) questionnaires^[8,9]^ were also administered at baseline to parents and young people, in English or Spanish, depending on individual preference. Young people aged 8−18 years completed the SEDM child version, whereas their parents completed the corresponding parent version. For the PAID, young people aged 8−12 years completed the child version, and those aged 13−18 years completed the teen version. Parents completed the PAID parent version (of children or teens, as appropriate) for all participants. Children younger than 8 years did not complete the PAID or SEDM surveys themselves; only the parent versions were completed in these cases.

During the course of the study, the study team recorded data about each study visit (video visit or RPM encounter) for participants in the intervention arms, including time spent, topics discussed (categorized as insulin adjustments, technology use, behavioral strategies, emotional support), and the clinician's assessment of whether the prior visit's recommendations had been implemented (categorized as all, some, or none). Determination of whether insulin dose recommendations had been implemented relied on a discussion with the patient and caregiver, as well as a review of most recent insulin pump settings for participants using pumps.

Study completion tasks (see paragraph below) were initiated at the 6-month mark for the usual care arm, and after completion of outreach events for the intervention arms, which sometimes extended slightly beyond 6 months in the case that video visits were rescheduled. Participants who chose not to continue receiving the intervention or were lost to follow-up were still included in the end-of-study data collection (e.g., HbA1c, healthcare use, surveys) if they were willing and if data could be obtained.

At time of study completion, repeat HbA1c data were collected either via in-office measurement if the participants were coming for scheduled in-person diabetes care, or via a mail-in home collection HbA1c assay. All end-of-study HbA1c measurements were collected within 1 month before or 2 months after the participant's study completion date. At study completion, the SEDM and PAID surveys were again administered to all participants, and the use of diabetes technology was again abstracted from the EHR. In addition, participants in the two intervention arms were surveyed regarding the perceived benefits and burdens of the intervention they received, and their preferences for future care. Completion of the HbA1c tests and psychosocial instruments at enrollment and study completion were incentivized with gift cards.

Healthcare utilization was assessed for all participants by extracting the dates of diabetes clinic visits (in-person and telehealth, excluding the supplemental video visits provided to participants in the video arm), emergency department (ED) visits, and hospitalizations for the 6 months prior to study enrollment and 6 months during participation in the study. Because our research team recruited study participants during their diabetes visits, making this visit a prerequisite for study enrollment, any diabetes visit associated with study enrollment (taking place on the day of or within the 4 weeks prior to the consent date) was excluded from the pre-study visit count.

### Analysis

The primary outcome for this study was HbA1c, with the secondary outcomes of diabetes technology use, healthcare utilization, and patient/parent-reported diabetes distress (via PAID) and self-efficacy (via SEDM). These outcomes were analyzed within and compared between the study arms via a modified intention-to-treat analysis, with each participant for whom data were available analyzed in their originally assigned arm. For the SEDM survey, a mean across-item score (on a scale of 1−10) was calculated for each participant at each time point. For the PAID survey, the total score for each participant at each timepoint was normalized to a scale of 100, given that the child, teen, parent of child, and parent of teen versions of the PAID survey each have different score totals.

Changes in mean HbA1c, mean number of diabetes visits, and mean SEDM and PAID scores were estimated within each study arm using a paired *t*-test. Between-arm differences in these outcomes at end of study were estimated using analysis of covariance (ANCOVA) to adjust for baseline values as well as patient-level covariates of age, gender, race/ethnicity (dichotomized as non-Hispanic White versus other), insurance type, and baseline pump use. Gender was designated according to the participants' self-reports, whereas race and ethnicity data were collected from the EHR. The proportions of participants in each arm using diabetes technology (CGM devices, insulin pumps) and the proportions requiring ED and hospital care were recorded but were not compared statistically because very low frequencies of participants who did not use diabetes technologies or who required emergency or hospital care.

Process data regarding the study visits (time spent, recommendations provided and used) were summarized to characterize the interventions delivered, and compared between the two intervention arms using the *t*-test or χ^2^-test, as appropriate. Perceived benefits and burdens of the interventions and preferences for future care were analyzed for each of the two intervention arms, and compared between these arms using the χ^2^-test.

## Results

### Study population

Eighty-two participants gave consent, of whom 65 were randomized after successful run-in; these 65 formed the cohort for our analyses. See Fig. 1 (CONSORT diagram) for additional details about the flow of participants through the study. In total, 45 participants completed the study; the remaining 20 chose not to continue with the intervention or were lost to contact prior to their 6-month completion date. Tragically, one participant in the usual care arm died during the study period, unrelated to their participation.

**Figure 1:**
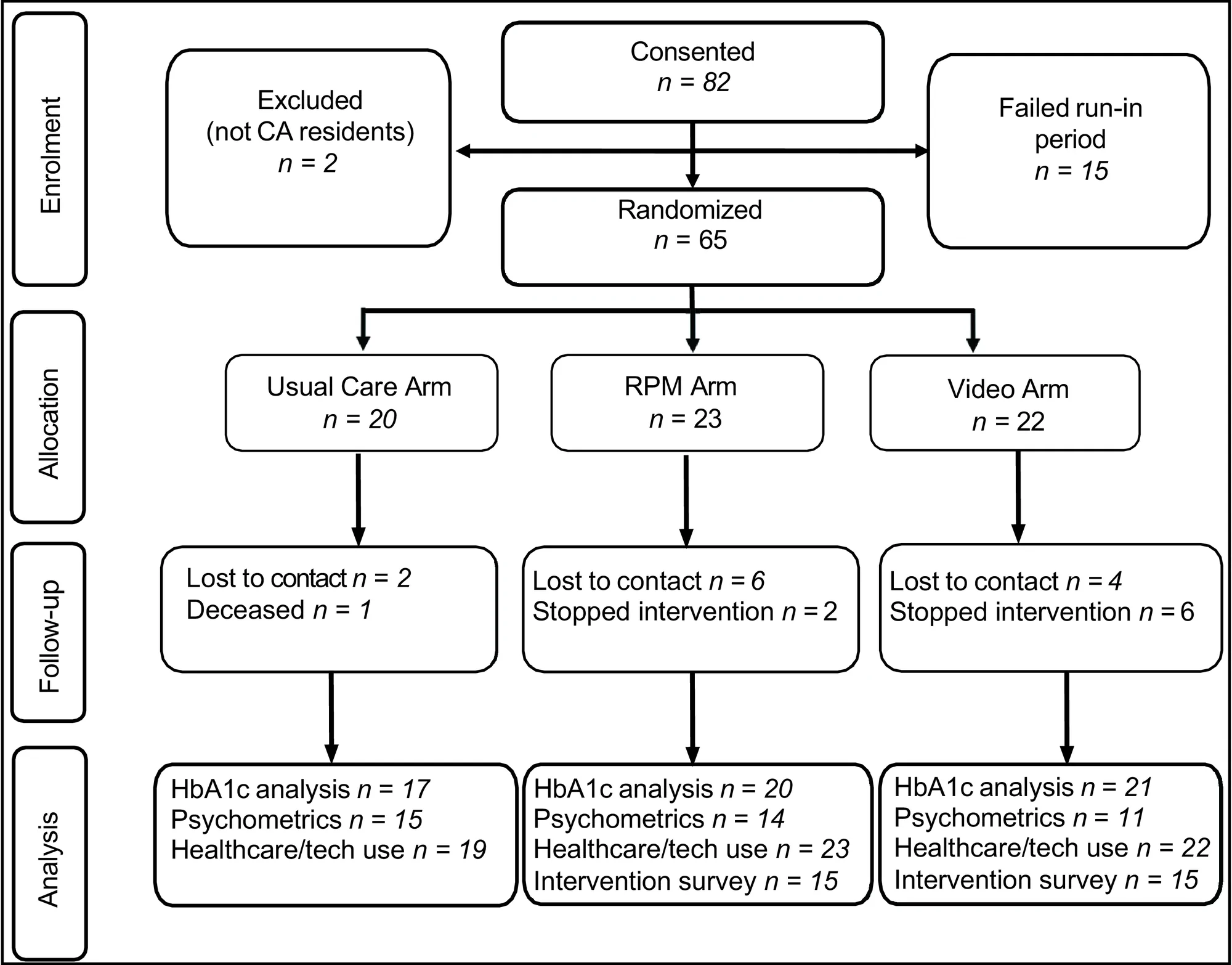
Consolidated Standards of Reporting Trials (CONSORT) diagram depicting study participation.

Of note, study attrition was not evenly distributed across the three groups, with 10 (45%) of the video arm participants, 8 (35%) of the RPM participants, and only 2 (10%), or 3 (15%) including the one deceased participant, of the usual care participants choosing not to receive the intervention or being lost to contact prior to study completion.

In total, 58 participants (89%) contributed to the final HbA1c analysis, 40 (62%) contributed to the psychosocial measures analyses, 64 (98%) contributed to the analyses of healthcare and technology use (the deceased participant did not have follow-up data for these), and 30 (67% of the 45 participants in the intervention arms) responded to the intervention survey.

The characteristics of the study population by assigned arm are shown in Table 1. Overall, our study population was 43% female, 26% Hispanic/Latino, 54% White, and 57% publicly insured, which reflects the overall demographics of our diabetes clinic. The mean age was 13.4 years, the mean T1D duration was 5.8 years, the mean baseline HbA1c was 9.7%, and the mean distance of the participant's home from clinic was 74 miles. Baseline CGM and pump use were 98.5% and 76.9%, respectively. The three study groups did not differ statistically on any of these measures.

**Table 1:** The study population's characteristics.

	Usual care (*n* = 20)	RPM (*n* = 23)	Video (*n* = 22)	Total (*N* = 65)	*p*-Value
Age (years), mean (SD)	14.6 (2.8)	12.5 (3.3)	13.2 (3.1)	13.4 (3.2)	0.112^1^
Female gender, *n* (%)	5 (25%)	14 (60.9%)	9 (40.9%)	28 (43.1%)	0.059^2^
Race, *n* (%)					0.670^2^
Asian	0 (0%)	1 (4.3%)	0 (0%)	1 (1.5%)	
Black or African American	2 (10%)	2 (8.7%)	4 (18.2%)	8 (12.3%)	
Other/unknown	3 (15%)	5 (21.7%)	7 (31.8%)	15 (23.1%)	
Two or more races	3 (15%)	2 (8.7%)	1 (4.5%)	6 (9.2%)	
White	12 (60%)	13 (56.5%)	10 (45.5%)	35 (53.8%)	
Hispanic/Latino ethnicity, *n* (%)	7 (35%)	3 (13%)	7 (31.8%)	17 (26.2%)	0.200^2^
Public insurance, *n* (%)	12 (60%)	10 (43.5%)	15 (68.2%)	37 (56.9%)	0.233^2^
Miles from clinic, mean (SD)	51.2 (54.9)	85.7 (84.9)	83.1 (104.6)	74.2 (84.8)	0.411^1^
T1D duration (years), mean (SD)	5.9 (4.3)	6.1 (4.2)	5.5 (3.6)	5.8 (4.0)	0.963^1^
CGM use, *n* (%)	20 (100%)	23 (100%)	21 (95.5%)	64 (98.5%)	0.371^2^
Insulin pump use, *n* (%)	15 (75%)	20 (87%)	15 (68.2%)	50 (76.9%)	0.318^2^
Baseline HbA1c (%), mean (SD)	9.9 (2.0)	9.9 (1.9)	9.4 (1.3)	9.7 (1.7)	0.690^1^
Baseline 30-day TIR (%), mean (SD)^3^	38.9 (14.1)	35.7 (15.3)	34.3 (12.5)	36.2 (13.9)	0.441^1^
Baseline pump TDD, mean (SD)^4^	1.0 (0.28)	1.1 (0.35)	1.0 (0.37)	1.0 (0.33)	0.445^1^
^1^ Kruskal–Wallis *p*-value; ^2^ χ^2^ *p*-value; ^3^ Data only available for *n* = 53; ^4^ Data only available for *n* = 37. SD, standard deviation; RPM, remote patient monitoring; T1D, type 1 diabetes; CGM, continuous glucose monitoring; HbA1c, hemoglobin A1c; TIR, time in range 70−180 mg/dL; TDD, total daily insulin dose in units per kg of body weight.					

### Intervention metrics

A comparison of clinician-reported metrics for the two interventions are shown in Table 2. Video visits were significantly longer (29.8 ± 10.9 min vs 17.2 ± 12.1 min for RPM, *p* < 0.001), and significantly more likely to cover topics besides insulin doses, specifically diabetes skills or behaviors (89% of video visits vs 68% of RPM encounters, *p* = 0.001), use of diabetes technology (78% of video vs 52% of RPM encounters, *p* < 0.001), and emotional support for managing diabetes (34% of video vs 10% of RPM encounters, *p* < 0.001). Recommendations provided during the video visits were also more likely to be used than those provided via asynchronous RPM outreach ("some" recommendations were implemented for 65% of video vs 42% of RPM encounters, and "none" were implemented for 22% of video vs 45% of RPM encounters, *p* = 0.008).

**Table 2:** Clinician-reported data from study encounters.

Visit metrics	Video	RPM	*p*-value^1^
Mean (SD), min	29.8 (10.9)	17.2 (12.1)	< 0.001
Topics covered			
Insulin doses	51%	55%	0.495
Diabetes skills/behaviors	89%	68%	0.001
Diabetes technology use	78%	52%	<0.001
Emotional support	34%	10%	<0.001
Recommendations used			0.008
All	13%	13%	
Some	65%	42%	
None	22%	45%	
^1^ *p*-values using the *t*-test or χ^2^-test, as appropriate			

The total intervention time received throughout the study differed significantly between the two intervention arms, with a mean (standard deviation [SD]) of 76 (51) min for RPM participants and 122 (67) min for video participants (*p* = 0.013). However, the total intervention time was not significantly associated with a change in HbA1c.

### Hemoglobin A1c

Analyses of within-arm changes and between-arm differences in HbA1c are shown in Table 3. In the single-arm unadjusted analysis, only the video arm demonstrated a significant improvement in mean HbA1c throughout the study (−0.53, 95% confidence interval [CI]: −0.93 to −0.12, *p* = 0.01). A comparison of the final HbA1c between the video and usual care arms, adjusting for baseline HbA1c, age, gender, race/ethnicity, insurance, and baseline pump use via ANCOVA, estimated a −1.0 mean HbA1c improvement for video vs usual care that was just shy of statistical significance (95% CI: −2.07 to 0.06, *p* = 0.06). The RPM arm did not demonstrate significant improvement in HbA1c from baseline to completion (−0.43, 95% CI: −1.23 to 0.38, *p* = 0.28) or as compared with usual care (−0.10, 95% CI: −1.21 to 1.04, *p* = 0.88). When multiple imputation was used to replace missing HbA1c values across all three study arms, the results were not substantively changed.

**Table 3:** Within-arm and between-arm comparisons of changes in continuous study outcomes.

		Measured mean HbA1c (%)	Mean diabetes visits in the prior 6 months	Mean SEDM^1^- youth score	Mean SEDM^1^- parent score	Mean PAID^2^- youth score	Mean PAID^2^- parent score
VIDEO	Baseline	9.40	1.68	6.10	7.80	38.50	39.50
	Final	8.80	1.36	6.70	8.20	38.00	39.60
	Δ	−0.53	−0.32	0.64	0.82	3.54	3.40
	(95% CI)	(−0.93, –0.12)	(−0.73, 0.10)	(−1.03, 2.30)	(−0.83, 2.48)	(−10.91, 17.98)	(−14.92, 21.71)
	*p*-value	0.01	0.13	0.41	0.30	0.60	0.69
	Outcome compared with usual care^3^	−1.00	−0.50	0.40	0.10	4.20	−1.40
	(95% CI)	(−2.07, 0.06)	(−1.05, 0.03)	(−0.82, 1.60)	(−1.15, 1.33)	(−13.72, 22.07)	(−20.57, 17.78)
	*p-*value	0.06	0.04	0.52	0.88	0.64	0.88
RPM	Baseline	9.90	1.70	6.30	7.80	37.20	47.90
	Final	9.60	1.96	6.70	7.80	30.90	49.30
	Δ	−0.43	0.26	0.56	−0.20	−6.70	0.28
	(95% CI)	(−1.23, 0.38)	(−0.16, 0.68)	(−0.35, 1.48)	(−0.94, 0.55)	(−18.62, 5.22)	(−10.49, 11.06)
	*p-*value	0.28	0.21	0.21	0.58	0.24	0.96
	Outcome compared with usual care^3^	−0.10	0.20	0.60	−0.30	−8.90	3.60
	(95% CI)	(−1.21, 1.04)	(−0.35, 0.74)	(−0.55, 1.79)	(−1.56, 0.89)	(−26.49, 8.66)	(−15.85, 23.14)
	*p-*value	0.88	0.47	0.29	0.58	0.31	0.71
Usual care	Baseline	9.90	1.55	6.30	8.00	47.50	49.10
	Final	9.80	1.74	6.50	8.40	33.10	42.70
	Δ	−0.05	0.21	0.15	0.56	−10.86	−8.39
	(95% CI)	(−0.81, 0.72)	(−0.44, 0.86)	(−0.78, 1.09)	(−0.12, 1.25)	(−22.40, 0.67)	(−20.54, 3.75)
	*p-*value	0.90	0.51	0.73	0.10	0.06	0.16
^1^ Mean per item score on a 10-point scale; ^2^ Total score on a 100-point scale; ^3^ ANCOVA comparison of the final values wth adjustment for baseline values, age, gender, race/ethnicity, insurance type, and baseline pump use.							

### Healthcare and technology utilization

CGM use was almost universal within our study population at baseline (20/20 for usual care, 23/23 for RPM, and 21/22 for video participants) and changed minimally during the study, with one usual care participant discontinuing its use and one video participant adopting CGM technology. Insulin pump use also did not change substantially from the baseline (15/20 for usual care, 20/23 for RPM, and 15/22 for video participants) to completion (15/20 for usual care, 20/23 for RPM, and 17/22 for video participants). With so few changes in use, between-arm differences in this outcome could not be statistically analyzed. Similarly, the frequency of ED and hospital encounters was very low in all three groups at baseline and completion (0−3 participants in usual care, 1−3 in RPM, and 4−6 in the video arm at both time points); therefore, a statistical comparison of differences across groups was not feasible.

As shown in Table 3, the mean number of diabetes clinic visits during the study was lower for the video arm compared with the usual care arm (*p* = 0.04), after adjusting for baseline visit frequency and other baseline factors (age, gender, race/ethnicity, insurance, pump use) via ANCOVA. In terms of raw counts, the video arm was the only arm in which the mean number of visits decreased (from 1.68 during the 6 months prior to 1.36 during the study period). This within-arm change was not statistically significant; however, the change in mean visit frequency was significantly different in comparison with other study arms because both the RPM and usual care arms had an increase in visit frequency during the study.

### Patient-reported outcomes

Analyses of within-arm changes and between-arm differences in SEDM and PAID scores for child and parent participants by study group are also shown in Table 3. Higher SEDM scores indicate greater self-efficacy in diabetes management; lower PAID scores indicate lower diabetes-related distress. Mean SEDM scores for parents and young participants in all three groups appeared to be stable (young people in the RPM arm) or to increase (all other groups) during the study, but none of the within-arm changes or between-arm comparisons for this measure were statistically significant. Changes in PAID scores varied significantly among participant groups. Interestingly, both parents and young people in the usual care arm had reductions in their mean PAID scores, and for young people, the change showed a trend toward significance (*p* = 0.06). There were no significant changes in mean PAID scores for video or RPM participants, or significant between-arm differences in the final PAID scores after adjusting for baseline variables.

### Feedback from intervention arms

A summary of participants' responses to the intervention survey administered to video and RPM arm participants at study completion is shown in Fig. 2. The majority of both RPM and video participants reported that their intervention was "very easy" and "very helpful", did not cost them any time, and was "definitely worthwhile".

**Figure 2:**
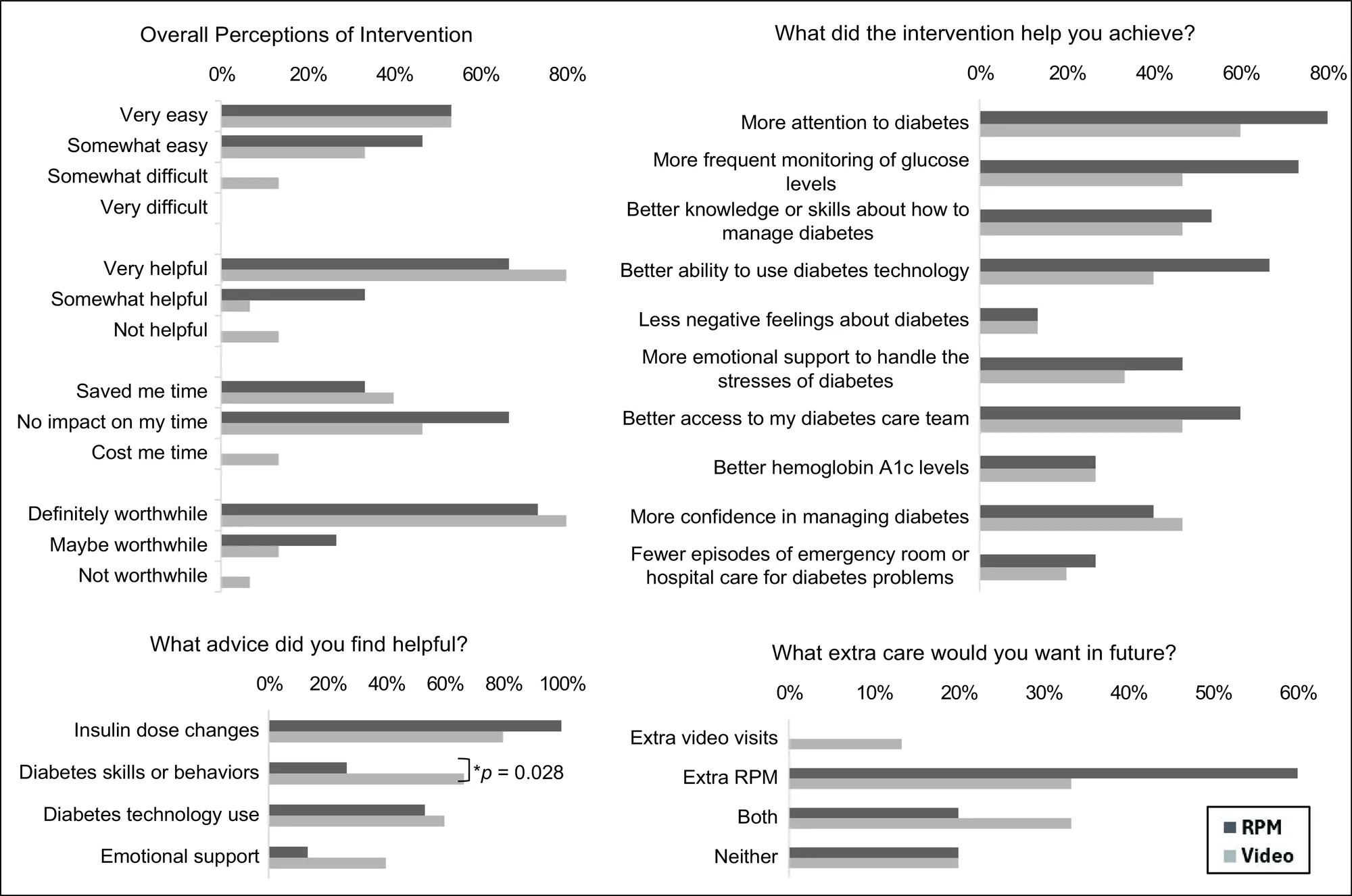
Participants' feedback about the RPM and video interventions. * Between-arm comparison was performed using the χ^2^-test for this and all other responses. No other *p*-values < 0.05.

When asked what aspects of the extra care they found most helpful, respondents from the two intervention arms differed slightly. All RPM respondents valued insulin dose adjustments and a majority (53%) also found advice about diabetes technology useful, but far fewer endorsed receiving helpful advice about diabetes skills or behaviors (27%) or emotional support for managing diabetes (13%). In contrast, video respondents also endorsed the utility of insulin dose changes (80%) and advice about diabetes technology (60%) but much higher proportions reported receiving helpful advice about diabetes skills or behaviors (67%) and emotional support for managing diabetes (40%). In this way, the participants' perceptions mirrored the clinicians' data, showing that a higher proportion of video visits touched on these latter two topic areas. The only comparison that was statistically significant between the two respondent groups was the perceived benefit from advice about diabetes skills or behaviors (67% vs 27%, *p* = 0.028).

Overall, respondents from both intervention arms endorsed a wide variety of perceived benefits from the extra care they received. For almost every category, a higher proportion of RPM than video participants reported receiving this benefit; the only exception was "more confidence in managing diabetes", which was endorsed by 47% of video respondents and 40% of RPM respondents. The most substantive differences in perceived benefits were for "more frequent monitoring of glucose levels" (73% RPM, 47% video), "better ability to use diabetes technology" (67% RPM, 40% video), and "more attention to diabetes" (80% RPM, 60% video). However, none of these differences was statistically significant.

Finally, when asked if they would choose to continue extra care in either form (RPM or video) moving forward, 80% of each group reported that they would like to continue, and more respondents from each group preferred RPM alone or RPM + video to video alone.

## Discussion

Our 6-month trial of monthly supplemental care via two contrasting modalities (video visits and asynchronous RPM) in a high-risk population (majority publicly insured, high baseline HbA1c) demonstrated high participant satisfaction with both modalities. The video arm demonstrated a statistically significant within-arm decrease in HbA1c (−0.53%, *p* = 0.01) and a clinically meaningful trend toward HbA1c improvement (−1.0%, *p* = 0.06) as compared with usual care, whereas the RPM arm did not experience any significant improvement in HbA1c.

Overall, the glycemic improvement seen in this study was smaller than expected on the basis of prior results. Given that the video arm had the highest attrition (45%), it is likely that our Intention To Treat (ITT) analysis did not capture the full efficacy of the video intervention; however, the attrition rate is an important finding regarding the feasibility of this intervention in a high-risk population. Our prior study of supplemental video visits was a single-arm trial and demonstrated a notable difference between HbA1c improvement in the intention-to-treat (0.8% reduction) versus the per-protocol analysis (1.2% reduction)^[4]^. Participants in that study also entered with a higher mean HbA1c (10.8% versus 9.4% in this study's video arm) and had lower baseline technology use (32% CGM and 44% pump versus 95% and 68%, respectively, for the video arm). This is important because another finding of our previous single-arm trial was the high adoption of technology. It is possible that increased technology use was a primary driver of the HbA1c changes experienced by participants, whereas the vast majority of our current study cohort was already using technology at the time of enrollment.

The RPM intervention in this trial did not appear to improve HbA1c among recipients or compared with usual care. The contrast between this (lack of) effect and the effect of video visits may relate to the differences observed between the two interventions: Video visits were longer, included more discussion of diabetes topics outside of insulin dose adjustments, and were more likely to result in the advice being used. However, a similar RPM intervention was previously effective at lowering GMI by 0.68% over 6 months among CGM users with baseline GMI ≥ 8%^[6]^. One possible explanation is the higher baseline use of pumps, all of which included automated insulin delivery (AID) systems, in this study's RPM participants (87% versus 74% pump use in the prior trial, none of which were AID systems). In light of this, the contrasting findings may be an indication that individuals using AID technology benefit less from frequent dose adjustments by clinicians, and the optimization of glycemic levels among those who have high HbA1c while on AID systems requires attention to the types of topics (e.g., diabetes self-management behaviors) that are better suited to a synchronous interactive encounter. Although total intervention time was not associated with change in HbA1c across the two intervention arms, the time spent during video visits reflected active engagement and involved the provision of broader and more personalized advice, which was likely crucial to its final effects on HbA1c.

Our trial showed no significant improvements in self-reported outcomes of self-efficacy or diabetes distress for either intervention arm. One factor may be that the baseline values for these measures were collected usually at the time of a diabetes visit, when concern about elevated HbA1c could strongly influence ratings of distress and self-efficacy, whereas the final values after 6 months were usually collected at a time between visits, when concerns about glycemic outcomes may have been less influential, particularly for usual care participants who were not receiving extra monthly outreach about their diabetes. Interestingly, responses to our intervention survey suggest that both interventions may have improved distress and self-efficacy for some, given that 13.3% of respondents in each group reported having "less negative feelings about diabetes", and 46.7% of video and 40% of RPM respondents reported "more confidence in managing diabetes" as a result of their intervention. It may be that the efficacy of these interventions to improve psychosocial aspects of living with diabetes is highly variable across individuals, and/or they primarily affect dimensions we did not assess, such as overall quality of life, illness perceptions, or relationships with or trust in healthcare providers.

Recipients of both interventions reported high satisfaction, but video visits were perceived overall as slightly less convenient, in keeping with the higher attrition rate for this study arm. This convenience factor may also explain why participants from both intervention arms reported preferring RPM to video as an extra care modality moving forward, despite video showing a trend toward greater efficacy for improving HbA1c in our analysis. It is notable that video arm participants attended clinic less often during the study, although fortunately not to such an extent that their overall care was less, since they also received monthly supplemental video visits. Further exploration is needed to conclude whether this small reduction in in-person visits represents a downside of the intervention (e.g., less engagement with the clinical care team), or improved convenience to families while maintaining adequate care.

There are several limitations of this study. First, it was unblinded, which may explain the high satisfaction and perceived benefits among the intervention participants even in the RPM arm, which did not appear to experience a quantifiable improvement. This may also have contributed to the lower clinic attendance observed among the video participants during the study (if they felt that they could postpone clinic visits because of receiving supplemental video care). However, the unblinded nature would not bias our analysis of the primary outcome (change in HbA1c) in this randomized controlled trial.

Another limitation is that we experienced a high attrition rate within our intervention groups, and this was higher among video participants than among RPM participants. This differential attrition seemed to directly relate to the time burden and degree of responsiveness required by the participants to remain in the study. For example, video participants needed to schedule and attend monthly video visits as part of the study, whereas RPM participants needed only to maintain data sharing and receive asynchronous messages from the study physician, and usual care participants did not have to complete any study-related tasks until the end of the study. The high level of attrition likely diluted our primary findings, since the analysis included many individuals who did not receive the full intervention. However, this attrition improves the external validity and generalizability of our findings, because it reflects the likely experience with the implementation of these interventions for similar populations in other settings. The participants were not paid to complete the study visits; therefore, the level of attrition and engagement seen in the study should reasonably reflect what would be seen in practice.

To better understand the factors influencing study attrition, we compared baseline characteristics between completers and noncompleters. We found that in addition to group assignment, noncompletion was associated with lower baseline time in range (TIR) (29.2 vs 38.7%, *p* = 0.012) and higher baseline total daily insulin dose (TDD) (1.3 vs 0.9 units/kg/day, *p* = 0.024) among those with available baseline CGM and pump data. This suggests that participants with more advanced or inadequately treated T1D at baseline were less likely to complete the study. However, no other baseline characteristics, including demographics and baseline HbA1c, were associated with completion status, so it is unclear if this association only holds true among those using diabetes technology.

Finally, a limitation of our glycemic analysis included the inability to utilize CGM metrics for analysis, in addition to HbA1c. Despite a high level of baseline technology use in our study population, we found that a low proportion of participants had CGM or pump data consistently available at all time points throughout the study. Video and RPM encounters often included assisting families with technology supply issues or data upload issues related to inconsistent cellular access, device connectivity, and account management. We observed a strong association between data availability and attention to diabetes care overall, suggesting that the available CGM data would be biased toward better glycemic metrics than the true glycemia experienced by the cohort as a whole. In addition, participants who did not continue with the interventions were not responsive to our attempts to restore data access, whereas we were able to obtain HbA1c data from the clinic visits they attended as part of usual care, making our HbA1c data far more complete. Given these factors and the high degree of missing CGM data, we could not analyze CGM metrics as a reliable reflection of glycemic changes for our study population.

Economics is an important consideration for any practice looking to provide supplemental care. In this study, monthly video and RPM encounters were not billed because they took place in a research context; in practice, both are reimbursable using existing Current Procedural Terminology (CPT) codes. As both interventions relied on prescribable glucose monitoring devices and freely available software platforms for remote data sharing, these prerequisites for diabetes-related telehealth care do not represent an added expense for practices or participants. Because we used telehealth for supplemental care rather than replacing quarterly diabetes visits, it did not offer time or money savings for families (i.e., through reductions in transportation costs or missed work). However, as our participants' survey responses demonstrate, they did not find either the video or RPM intervention burdensome. Therefore, although a formal economic analysis was not feasible for either study intervention with our available data, it appears to be economically viable for both practices and patients to implement supplemental diabetes-related telehealth care via video and/or asynchronous RPM.

## Conclusions

This randomized controlled trial adds to our understanding of the effectiveness of supplemental care for PwT1D with elevated glycemic levels in several ways. First, it demonstrates that among patients with elevated glycemic levels in the setting of established T1D and ongoing AID use, synchronous supplemental care that touches on topics of diabetes skills and behaviors, emotional support, and optimizing technology use may be more effective at improving glycemic levels than supplemental asynchronous RPM based on glycemic data alone. However, it also illustrates that supplemental monthly video visits require a significant time commitment (122 ± 67 minutes over 6 months) and are not acceptable to a subset of individuals and families in this target population, as exhibited by our high attrition rate (45%) within the video arm. Finally, it shows that PwT1D and their families in this cohort tend to be highly appreciative of receiving either synchronous or asynchronous supplemental care, and to find this extra care worthwhile. Given our findings that synchronous video visits focused on skills, behaviors, and emotional support may be most effective at improving outcomes for this high-risk population, future studies should explore whether more frequent contact with a certified diabetes care and education specialist or a behavioral specialist can achieve equivalent or better outcomes than monthly endocrinologist outreach in this group.

## Data Availability

Deidentified data from this study can be accessed at clinicaltrials.gov (NCT04428658).
